# Association between serum phospholipid fatty acid levels and adiposity in Mexican women[S]

**DOI:** 10.1194/jlr.P073643

**Published:** 2017-05-02

**Authors:** Elom K. Aglago, Carine Biessy, Gabriela Torres-Mejía, Angélica Angeles-Llerenas, Marc J. Gunter, Isabelle Romieu, Veronique Chajès

**Affiliations:** Nutrition and Metabolism Section,* International Agency for Research on Cancer (IARC/WHO), Lyon, France; National Institute of Public Health,† Center for Population Health Research, Cuernavaca Morelos, Mexico

**Keywords:** obesity, desaturation index, body mass index, waist-to-hip ratio.

## Abstract

Fatty acids (FAs) have been postulated to impact adiposity, but few epidemiological studies addressing this hypothesis have been conducted. This study investigated the association between serum phospholipid FAs (S-PLFAs) and indicators of obesity. BMI and waist-to-hip ratio (WHR) were collected from 372 healthy Mexican women included as controls in a case-control study. S-PLFA percentages were determined through gas chromatography. Desaturation indices, SCD-16, SCD-18, FA desaturase (FADS)1, and FADS2, biomarkers of endogenous metabolism, were proxied respectively as 16:1n-7/16:0, 18:1n-9/18:0, 20:4n-6/20:3n-6, and 22:6n-3/20:5n-3. Multiple linear regressions adjusted for relevant confounders and corrected for multiple testing were conducted to determine the association between S-PLFA, desaturation indices, and indicators of adiposity. SCD-16 (β = 0.034, *P* = 0.001, q = 0.014), palmitoleic acid (β = 0.031, *P* = 0.001, q = 0.014), and dihomo-γ-linolenic acid (β = 0.043, *P* = 0.000, q = 0.0002) were positively associated with BMI. Total n-6 PUFAs (β = 1.497, *P* = 0.047, q = 0.22) and the ratio of n-6/n-3 PUFAs (β = 0.034, *P* = 0.040, q = 0.19) were positively associated with WHR, while odd-chain FAs (pentadecanoic and heptadecanoic acid) showed negative associations with all the adiposity indicators. In conclusion, increased endogenous synthesis of palmitoleic acid and a high n-6/n-3 ratio are associated with increased adiposity, while odd-chain FAs are associated with decreased adiposity. Further studies are needed to determine the potential causality behind these associations.

The obesity pandemic with associated comorbidities, such as respiratory and cardiovascular diseases, cancers, and other chronic diseases, is expanding worldwide (1). The situation is worsening in the developing world, with countries such as Mexico exhibiting a concerning proportion of obese individuals (2). According to recent estimates, approximately 65.3% of Mexican women are overweight or obese (1). Further, according to recent projections, more than 90% of Mexican women are at risk of becoming overweight or obese by 2050, with profound consequences on noncommunicable disease incidence and placing greater burden on the health system (3).

The global increase in obesity is predominantly attributed to the so-called obesogenic environment, which encompasses an increase in dietary energy intake and higher intake of added monosaccharides and saturated and *trans* fats, along with an unprecedented shift in energy expenditure patterns with a global decrease in physical activity and an increase in sedentary behaviors (4). The introduction of fast-food chains and the dissemination of Westernized dietary habits seem to be a marker of the increasing prevalence of obesity (5).

Several epidemiological studies have examined the relationship between dietary fat and FAs estimated through dietary questionnaires and obesity, but the epidemiological evidence remains insufficient. Melanson, Astrup, and Donahoo (6) summarized interventional prospective cohorts and cross-sectional studies and reported mitigated associations, although increased intakes of saturated FAs (SFAs), MUFAs, and *trans* FAs (TFAs) were associated with slightly higher risk of obesity, while a minor protective effect was reported for PUFAs.

Nutritional epidemiology is limited by the assessment of dietary FAs through food-frequency questionnaires or other dietary assessment methods (7). FA content of adipose tissue, triacylglycerol, phospholipid, and cholesterol ester fractions of serum, plasma, or erythrocyte membranes reflect long-term to short-term intake of dietary FAs (8). As the half-life of FAs in adipose tissue is 2 years long, adipose tissue FA composition can provide major information about long-term intake (9). However, most ongoing epidemiological studies have available blood fractions instead of adipose tissue samples. Triacylglycerol fractions in blood are influenced by the type and amount of fat consumed during recent meals; thus, they are not the most appropriate markers of usual diet. The FA composition of serum, plasma, or erythrocyte membrane phospholipid fraction reflects medium-term (weeks to months) intakes of some FAs or food sources, particularly for FAs that are not endogenously synthesized, industrial TFAs (ITFAs) and n-3 PUFAs (8, 10, 11). Besides FAs derived from dietary sources, desaturation indices can be determined in plasma or serum phospholipids as the ratio of products to substrates and are used as surrogate biomarkers of endogenous lipogenesis of FAs (12). Thus, serum (or plasma) phospholipid FA (S-PLFA) composition can be considered as a convenient alternative for the assessment of medium-term usual intake and metabolism of FAs in large-scale epidemiologic studies (13–16).

Few studies have investigated the association between biomarkers of FAs, FA metabolism, and obesity. One study conducted within the European EPIC cohort has reported an association between high percentages of plasma phospholipid ITFAs and increased risk of weight gain during the follow-up, particularly in women (17). A recent review by Mika and Sledzinski (18) reported a trend for a positive association between obesity and serum SFAs, MUFAs, and TFAs, and a negative association for odd-chain and branched SFAs. Endogenous production of FAs through the desaturation activity influences the relative levels of individual S-PLFAs. The desaturation activity is surrogated with the desaturation indices, SCD-16 and SCD-18, for the conversion of SFAs into MUFAs, and FA desaturase (FADS)1 and FADS2 for the introduction of additional double bonds to n-3 and n-6 PUFAs. SCD-16 has been reported to be positively associated with obesity indicators and body fatness, while the associations are mitigated for SCD-18 in cross-sectional studies (19–21). There is a competition for the desaturation of n-3 and n-6 PUFAs, resulting in probably differential association with obesity depending on n-6 and n-3 levels (22). Overall, there is a dearth of information regarding the association between FA biomarkers, endogenous FA synthesis, and obesity.

The aim of this study was to determine the association between S-PLFA percentages as biomarkers of dietary exposure and endogenous FA metabolism and indicators of obesity in a population of Mexican women. The novelty of this study relies on the wide range of FAs measured in serum phospholipids as biomarkers of dietary FAs and their endogenous metabolism.

## MATERIALS AND METHODS

### Study population

The population study was comprised of a subgroup of women enrolled as controls (non-cancer cases) in a population-based case-control study on breast cancer conducted in Mexican women from 2004 to 2007, as previously described (23). Briefly, participants in the current study were recruited from Mexico City, Monterrey, and Veracruz and matched to newly diagnosed breast cancer patients on the basis of age, region, and healthcare provider.

### Questionnaires

An individual questionnaire was completed for each participant at the recruitment. The questionnaire contained information on the participant’s socio-economic and demographic status, education history, self-reported personal and family morbidity (e.g., diabetes, hypertension, and arthritis), reproductive factors (gynecological history, menorrhea, contraception, and pregnancy), physical activity, smoking status, and dietary consumption.

Menopausal status was defined according to the halting of the amenorrhea for 12 consecutive months, at least. In contrast to premenopausal women, postmenopausal women were those with natural menopause (over 12 months cessation of menstruation) and those with surgical menopause, who reported bilateral oophorectomy or those who did not know the type of surgery, but were over 48 years, given that mean age at menopause in Mexican women is 48 years (24). Age at first menses and menarche were also collected.

Physical activity was assessed considering the duration (minutes), the motive (occupation, exercise, leisure), the time (morning, evening, night), and the intensity (light, moderate, and vigorous) of the activities executed (e.g., walking, gardening, writing, watching television, reading a book, karate, etc.) and was reported in total metabolic equivalent of task (MET) hours per week. Smoking status considered self-reported current, never, or former smoking status.

Energy intake was determined from a dietary food frequency questionnaire of 12 months previously validated in the Mexican population (25). Alcohol intake was computed from alcohol type consumed, as well as frequency of the consumption provided in the food frequency questionnaire.

### Anthropometry

Anthropometric indicators were measured by trained field workers using standardized international recommendations (26). Weight (kilograms) and height (centimeters) were measured using, respectively, an electronic scale and a stadiometer, with the women lightly dressed, barefoot, and in the orthostatic position. Waist (centimeters) and hip (centimeters) circumferences were measured with the woman in the standing position using a graduated, nonextensible tape, held not constricting, parallel to the floor, at the midpoint between the lowest rib and the iliac crest for the waist, and at the prominent part of the buttocks for the hip. BMI (weight/height squared, kilograms per square meter) and waist-to-hip ratio (WHR; waist/hip) were calculated using these measurements. BMI categories for underweight, normal or healthy weight, overweight, obesity, and morbid obesity were respectively <18.5 kg/m^2^, 18.5–24.9 kg/m^2^, 25–29.9 kg/m^2^, 30–39.9 kg/m^2^, and from and above 40 kg/m^2^.

Body silhouettes were recalled as subjective measurements using a pictographic chart composed of six women corpulence drawings at incremental sizes hypothetically from leanness to morbid obesity (1, underweight; 2 and 3, normal weight; 4, overweight; 5, obesity; 6, morbid obesity) (27). Participants were asked to select the silhouette they most closely resembled during childhood (6–11 years), adolescence (12–18 years), early adulthood (18–20 years), later adulthood (25–35 years), and at the time of enrolment in the study.

### Ethical clearance

Study participants provided written informed consent to participate in the study. Ethical approval was obtained from the Institutional Review Board of the Mexican National Institute of Public Health and from each of the participating hospitals.

### Analysis of S-PLFA

Fasting blood samples were collected by venepuncture at recruitment, centrifuged, aliquoted and stored at −80°C. S-PLFA concentration was measured using the lipidomic gas chromatography platform at the International Agency for Research on Cancer (Lyon, France). The methodology allows the separation and quantification of 60 FAs, including 15 TFA isomers from industrial processing and natural animal sources in a 1 h run.

Briefly, total lipids were extracted from serum samples (200 μl) with chloroform-methanol 2:1 (v/v) containing antioxidant butylated hydroxytoluene and L-A-phosphatidylcholine-dimyristoyl-d_54_ as an internal standard. Phospholipids were purified by adsorption chromatography. FA methyl esters (FAMEs) were formed by transmethylation with Methyl-Prep II (Alltech, Deerfield, IL). Analyses were carried out on the gas chromatograph 7890A (Agilent Technologies, Santa Clara, CA). Select for FAME capillary columns (Agilent Technologies), which have the specificity to discriminate TFAs from *cis* FAs, were used for separation of FAMEs. Each FA was identified with purchased standards, which were injected in the gas chromatograph to determine their retention time.

The relative levels of each FA, expressed as percent of total FAs, were quantified by integrating the area under the peak and dividing the result by the total area. Coefficients of variation for FAs ranged from 1.81% for large peaks (peak >1% of total FAs) to 9.75% for the smallest peaks (peak <1% of total FAs). Using values for 60 individual FAs, we calculated the percentage of the following groups: SFAs, *cis*-MUFAs, ruminant TFAs, ITFAs, which are TFA isomers obtained during the industrial processing of vegetal fats by heating, refining, or hydrogenation, *cis*-n-6 PUFAs, long-chain n-6 PUFAs, n-3 PUFAs, and long-chain n-3 PUFAs. We calculated the ratio of long-chain n-6/long-chain n-3 PUFAs. We also determined the desaturation indices as the ratio of product to substrate, either oleic acid to stearic acid (SCD-18) or the ratio of palmitoleic acid to palmitic acid (SCD-16), as biomarkers of stearoyl-CoA desaturase activity (12). In addition, the activity of FADSs, FADS1 and FADS2, were determined by the ratio of 20:4n-6 [arachidonic acid (AA)] to 20:3n-6 [dihomo-γ-linolenic acid (DGLA)] and 22:6n-3 (DHA) to 22:5n-3 [docosapentaenoic acid (DPA)], respectively.

### Statistical analyses

FAs expressed as the percentage of total FAs were used for the statistical analysis. Frequencies were presented for the categorical variables, while means and SDs were presented for all the continuous variables. FA values were log-transformed and geometric means with 95% CI were reported for the analysis.

Multiple linear regressions using the FAs or the desaturation indices as dependent variables and obesity indicators (BMI, waist, WHR) as independent variables were performed. Prior to the regression analysis, FA levels were Z-standardized for better comparison. Regression adjustments were performed for age (continuous variable), menopausal status (pre- and postmenopause), physical activity (total MET in minutes per week), smoking status (nonsmokers, current smokers, exsmokers), education (none, incomplete primary, complete primary, complete secondary, com­plete high school), alcohol consumption (grams per day), energy intake (kilocalories per day), and analytical batch. The coefficient (β), the SE, and the *P* value of the multiple regressions were reported. Due to the number of tests performed, q-values were calculated by transforming the *P* values for multiple comparisons using the false discovery rate via the Benjamini-Hochberg procedure (28). Statistical analyses were performed using STATA version 14.1 (StataCorp, College Station, TX) and R (R Foundation for Statistical Computing version 3.0.2, Vienna, Austria).

## RESULTS

The general characteristics of the study population are presented in **Table 1**. A total of 372 women with an average age of 50.1 years at the time of recruitment were included in the study. The prevalence of overweight and obesity in the population study were respectively 43.0% and 45.7% with a mean BMI of 30.3 kg/m^2^. Median silhouette shape increased throughout life course from level 1 at childhood to reach level 4 at adulthood (*P* = 0.011), showing an evolution from leanness to obesity with age in the study population.

**TABLE 1.** General characteristics of the study population

Variables	Mean ± SD or Frequency (%)
Age, years	50.1 ± 9.5
Weight, kg	69.9 ± 13.5
Height, cm	151.8 ± 6.0
BMI, kg/m^2^	30.3 ± 5.2
Waist, cm	98.2 ± 12.6
Hip, cm	108.2 ± 11.3
WHR	0.91 ± 0.06
Age at first menarche, years	12.9 ± 1.6
Age at menopause, years	48.3 ± 5.8
Physical activity, total MET min/week	276.3 ± 47.1
Alcohol intake, g/week	5.1 ± 29.7
Energy intake, kcal/day	1,921.6 ± 705.3
Menopause, %	174 (46.8)
Smokers, %	108 (29.0)
Diabetes, %	51 (13.7)
Education, %	
None	28 (7.5)
Incomplete primary	92 (24.7)
Complete primary	119 (32.0)
Complete secondary	100 (26.9)
Complete high school	17 (4.6)
College or graduate education	16 (4.3)
Silhouette at childhood (6–11 years)	
1*^a^*	206 (55.4)
2	101 (27.2)
3	50 (13.4)
4	10 (2.7)
5	5 (1.3)
6	0
Silhouette at adolescence (12–18 years)	
1	138 (37.1)
2*^a^*	145 (39.0)
3	67 (18.0)
4	15 (4.0)
5	5 (1.3)
6	2 (0.5)
Silhouette at early adulthood (18–20 years)	
1	67 (18.0)
2*^a^*	156 (41.9)
3	115 (30.9)
4	25 (6.7)
5	5 (1.3)
6	4 (1.1)
Silhouette at adulthood (25–35 years)	
1	15 (4.0)
2	94 (25.3)
3*^a^*	145 (39.1)
4	90 (24.3)
5	24 (6.5)
6	3 (0.8)
Silhouette actual (at the time of the recruitment)	
1	6 (1.6)
2	35 (9.4)
3	88 (23.7)
4*^a^*	149 (40.2)
5	62 (16.7)
6	31 (8.4)

Missing values: one for silhouette at adulthood and actual silhouette; four for weight, height, BMI, and waist; five for hip and WHR; 15 for alcohol consumption.

a Median value.

The S-PLFA profile of the population is indicated in **Table 2**. Individual FAs are grouped by family (saturates, monounsaturates, n-6 and n-3 polyunsaturates) and by conformation (*trans* and *cis*). The relative amount (percent of total FAs) of groups (SFAs, MUFAs, ruminant TFAs, ITFAs, n-3 and n-6 PUFAs) and the n-6/n-3 PUFA ratio were provided. Desaturation indices, SCD-16, SCD-18, FADS1, and FADS2, used as biomarkers of FA metabolism were also provided. Palmitic acid (16:0) and linoleic acid (18:2n-6) were the most abundant S-PLFAs, accounting for the high percentages of total SFAs and n-6 PUFAs. Compared with n-6 PUFAs, n-3 PUFA percentages were lower and this was reflected in an n-6/n-3 PUFA ratio of 8.2. ITFAs represented 1.4% of total S-PLFAs.

**TABLE 2.** Geometric means of S-PLFAs and desaturation indices in the study population

	Mean (95% CI)
SFAs	
14:0 (myristic acid)	0.20 (0.19–0.21)
15:0 (pentadecanoic acid)	0.14 (0.13–0.14)
16:0 (palmitic acid)	23.8 (23.6–24.1)
17:0 (heptadecanoic acid)	0.38 (0.38–0.39)
18:0 (stearic acid)	14.7 (14.5–14.9)
MUFAs	
* cis*-MUFA	
16:1n-7 (palmitoleic acid)	0.68 (0.66–0.70)
18:1n-5	0.21 (0.20–0.22)
18:1n-7 (cis-vaccenic acid)	1.48 (1.45–1.50)
18:1n-9 (oleic acid)	10.2 (10.1–0.4)
*trans*-MUFA	
16:1n-7/9 (palmitelaidic acid)	0.58 (0.55–0.61)
18:1n-9/12 (elaidic acid)	0.51 (0.49–0.53)
18:1n-7 (vaccenic acid)	0.18 (0.17–0.19)
PUFAs	
*cis*-n-6 PUFA	
18:2n-6 (linoleic acid)	24.0 (23.7–24.3)
18:3n-6 (γ-linolenic acid)	0.10 (0.09–0.10)
20:3n-6 (DGLA)	3.95 (3.86–4.04)
20:4n-6 (AA)	10.4 (10.2–10.6)
22:4n-6 (adrenic acid)	0.50 (0.49–0.51)
22:5n-6 (osbond acid)	0.45 (0.44–0.46)
*trans*-n-6 PUFA	
Conjugated linoleic acid	0.19 (0.18–0.19)
18:2ct, 18:2tc, 18:2tt (*trans* linoleic acid)	0.16 (0.15–0.16)
*cis*-n-9 PUFA	
20:3n-9 (mead acid)	0.12 (0.12–0.13)
*cis*-n-3 PUFA	
18:3n-3ccc (α-linolenic acid)	0.27 (0.26–0.28)
20:5n-3 (EPA)	0.46 (0.44–0.48)
22:5n-3 (DPA)	0.93 (0.91–0.95)
22:6n-3 (DHA)	3.10 (3.03–3.17)
*trans*-n-3 PUFA	
18:3n-3cct, ctt, ttt (*trans* α-linolenic acid)	0.07 (0.07–0.08)
Groupings	
Total SFAs	39.5 (39.2–39.8)
Total *cis*-MUFAs	13.0 (12.8–13.1)
Total *trans* ruminant FAs	0.39 (0.38–0.41)
Total ITFAs	1.39 (1.35–1.43)
Total *cis*-n-6 PUFAs	40.3 (40.0–40.6)
Total long-chain n-6 PUFAs	16.0 (15.8–16.2)
Total *cis*-n-3 PUFAs	4.91 (4.82–5.01)
Total long-chain n-3 PUFAs	4.63 (4.53–4.72)
Ratio PUFA n-6/n-3	8.19 (8.03–8.36)
Desaturation indexes	
SCD-16 (16:1n-7cis/16:0)	0.03 (0.027–0.029)
SCD-18 (18:1n-9cis/18:0)	0.70 (0.68–0.71)
FADS1 (20:4n-6/20:3n-6)	2.63 (2.55–2.71)
FADS2 (DHA/EPA)	6.72 (6.40–7.06)

FA values are expressed as percentage of total FAs. The values provided are geometric means with 95% CI.

**Table 3** shows the multiple regressions for the association between individual FAs and subgroups, and BMI, waist circumference, and WHR. Prior to control for multiple testing, BMI was positively associated with stearic acid (β = 0.024, *P* = 0.007), palmitoleic acid (β = 0.031, *P* = 0.001), DGLA (β = 0.043, *P* = 0.000), and EPA (β = 0.025, *P* = 0.020), and negatively associated with heptadecanoic acid (β = −0.026, *P* = 0.009) and *cis*-vaccenic acid (β = −0.027, *P* = 0.007). When the associations were corrected for multiple testing, q-values remained significant for palmitoleic acid (q = 0.014) and DGLA (q = 0.0002). Similar results were obtained for waist circumference. In contrast, the odd-chain FAs, pentadecanoic acid (β = −1.733, *P* = 0.012, q = 0.079) and heptadecanoic acid (β = −3.778, *P* = 0.000, q = 0.0002), tended to be negatively associated with WHR, while n-6 PUFAs (β = 1.497, *P* = 0.047, q = 0.22) and the n-6/n-3 PUFA ratio (β = 1.654, *P* = 0.040, q = 0.19) showed a positive trend with WHR. Finally, we did not observe a significant positive trend between ITFAs and obesity.

**TABLE 3.** Multiple regression models for the association between S-PLFAs and obesity indicators

	BMI				Waist Circumference				WHR			
	β*^a^*	SE	*P*	q	β*^a^*	SE	*P*	q	β*^a^*	SE	*P*	q
SFAs												
14:0 (myristic acid)	0.008	0.008	0.31	0.70	0.004	0.003	0.21	0.55	1.237	0.630	0.051	0.22
15:0 (pentadecanoic acid)	0.001	0.009	0.93	0.98	−0.005	0.004	0.16	0.47	−1.733	0.684	**0.012**	0.079
16:0 (palmitic acid)	0.004	0.007	0.56	0.87	0.001	0.003	0.68	0.95	0.352	0.565	0.53	0.86
17:0 (heptadecanoic acid)	−0.026	0.010	**0.009**	0.065	−0.017	0.004	**0.000**	**0.0002**	−3.778	0.776	**0.000**	**0.0002**
18:0 (stearic acid)	0.024	0.009	**0.007**	0.058	0.005	0.004	0.19	0.52	−0.926	0.701	0.19	0.52
MUFAs												
*cis*-MUFAs												
16:1n-7 (palmitoleic acid)	0.031	0.010	**0.001**	**0.014**	0.012	0.004	**0.003**	**0.033**	1.220	0.769	0.11	0.39
18:1n-5	−0.005	0.010	0.60	0.89	−0.002	0.004	0.67	0.95	−0.967	0.790	0.22	0.56
18:1n-7 (cis-vaccenic acid)	−0.027	0.010	**0.007**	0.058	−0.010	0.004	**0.012**	0.079	−1.896	0.787	**0.017**	0.11
18:1n-9 (oleic acid)	−0.018	0.010	0.069	0.27	−0.008	0.004	0.052	0.22	−0.457	0.766	0.55	0.87
*trans*-MUFAs												
16:1n-7/9 (palmitelaidic acid)	0.005	0.010	0.54	0.86	0.001	0.003	0.79	0.97	−0.134	0.605	0.82	0.97
18:1n-9/12 (elaidic acid)	−0.013	0.010	0.21	0.55	−0.003	0.004	0.49	0.85	−0.405	0.798	0.61	0.90
18:1n-7 (vaccenic acid)	−0.010	0.008	0.34	0.71	−0.003	0.003	0.35	0.71	−0.897	0.652	0.17	0.48
PUFAs												
*cis*-n-6 PUFAs												
18:2n-6 (linoleic acid)	−0.007	0.010	0.47	0.84	0.003	0.004	0.41	0.76	1.54	0.799	0.055	0.23
18:3n-6 (γ-linolenic acid)	0.027	0.010	**0.009**	0.065	0.004	0.004	0.36	0.72	0.014	0.811	0.99	0.99
20:3n-6 (DGLA)	0.043	0.010	**0.000**	**0.0002**	0.013	0.004	**0.002**	**0.023**	0.624	0.805	0.44	0.79
20:4n-6 (AA)	−0.007	0.010	0.52	0.86	−0.004	0.004	0.29	0.69	−0.550	0.810	0.50	0.86
22:4n-6 (adrenic acid)	−0.007	0.010	0.49	0.85	−0.003	0.004	0.51	0.86	0.952	0.800	0.23	0.58
22:5n-6 (osbond acid)	−0.029	0.010	**0.006**	0.053	−0.010	0.004	**0.019**	0.12	0.801	0.823	0.33	0.71
*trans*-n-6 PUFAs												
Conjugated linoleic acid	0.014	0.010	0.16	0.47	0.006	0.004	0.13	0.42	0.330	0.791	0.68	0.95
18:2ct, 18:2tc, 18:2tt (*trans* linoleic acid)	0.001	0.009	0.92	0.98	0.002	0.004	0.60	0.89	0.191	0.685	0.78	0.97
*cis*-n-9 PUFAs												
20:3n-9 (mead acid)	0.011	0.010	0.29	0.69	0.001	0.004	0.78	0.97	−0.068	0.796	0.93	0.98
*cis*-n-3 PUFAs												
18:3n-3ccc (α-linolenic acid)	−0.005	0.011	0.61	0.90	−0.001	0.004	0.74	0.97	−0.283	0.847	0.74	0.97
20:5n-3 (EPA)	0.025	0.010	**0.020**	0.12	0.007	0.004	0.12	0.40	−0.461	0.82	0.57	0.87
22:5n-3 (DPA)	−0.005	0.010	0.59	0.89	−0.001	0.004	0.88	0.97	0.013	0.781	0.99	0.99
22:6n-3 (DHA)	−0.009	0.010	0.37	0.74	−0.006	0.004	0.17	0.48	−1.312	0.792	0.098	0.35
*trans*-n-3 PUFAs												
18:3n-3cct, ctt, ttt (*trans* α-linolenic acid)	0.001	0.010	0.94	0.98	0.003	0.004	0.53	0.86	0.795	0.829	0.34	0.71
Groupings												
Total SFAs	0.018	0.009	0.051	0.22	0.003	0.004	0.38	0.74	−0.429	0.708	0.54	0.86
Total *cis*-MUFAs	−0.017	0.010	0.07	0.27	−0.007	0.004	0.057	0.24	−0.633	0.757	0.40	0.76
Total *trans* ruminant FAs	−0.002	0.009	0.83	0.97	−0.001	0.004	0.73	0.97	−0.734	0.710	0.30	0.70
Total ITFAs	−0.004	0.010	0.67	0.95	−0.001	0.004	0.84	0.97	−0.231	0.729	0.75	0.97
Total *cis*-n-6 PUFAs	−0.002	0.010	0.86	0.97	0.004	0.004	0.31	0.70	1.497	0.752	**0.047**	0.22
Total long-chain n-6 PUFAs	0.008	0.010	0.41	0.76	0.001	0.004	0.89	0.97	−0.113	0.781	0.88	0.97
Total *cis*-n-3 PUFAs	−0.002	0.010	0.80	0.97	−0.003	0.004	0.49	0.85	−1.129	0.784	0.15	0.47
Total long-chain n-3 PUFAs	−0.002	0.010	0.87	0.97	−0.002	0.004	0.53	0.86	−1.080	0.783	0.17	0.48
Ratio PUFA n-6/n-3	0.002	0.010	0.86	0.97	0.004	0.004	0.31	0.70	1.654	0.803	**0.040**	0.19

a FA values were log-transformed and Z-standardized for the analysis for better comparison. All the regression models were adjusted for age, alcohol consumption, smoking, energy intake, education, physical activity, menopause, and batch of analysis. *P* or *q* values < 0.05 are shown in boldface type.

**Fig. 1.**
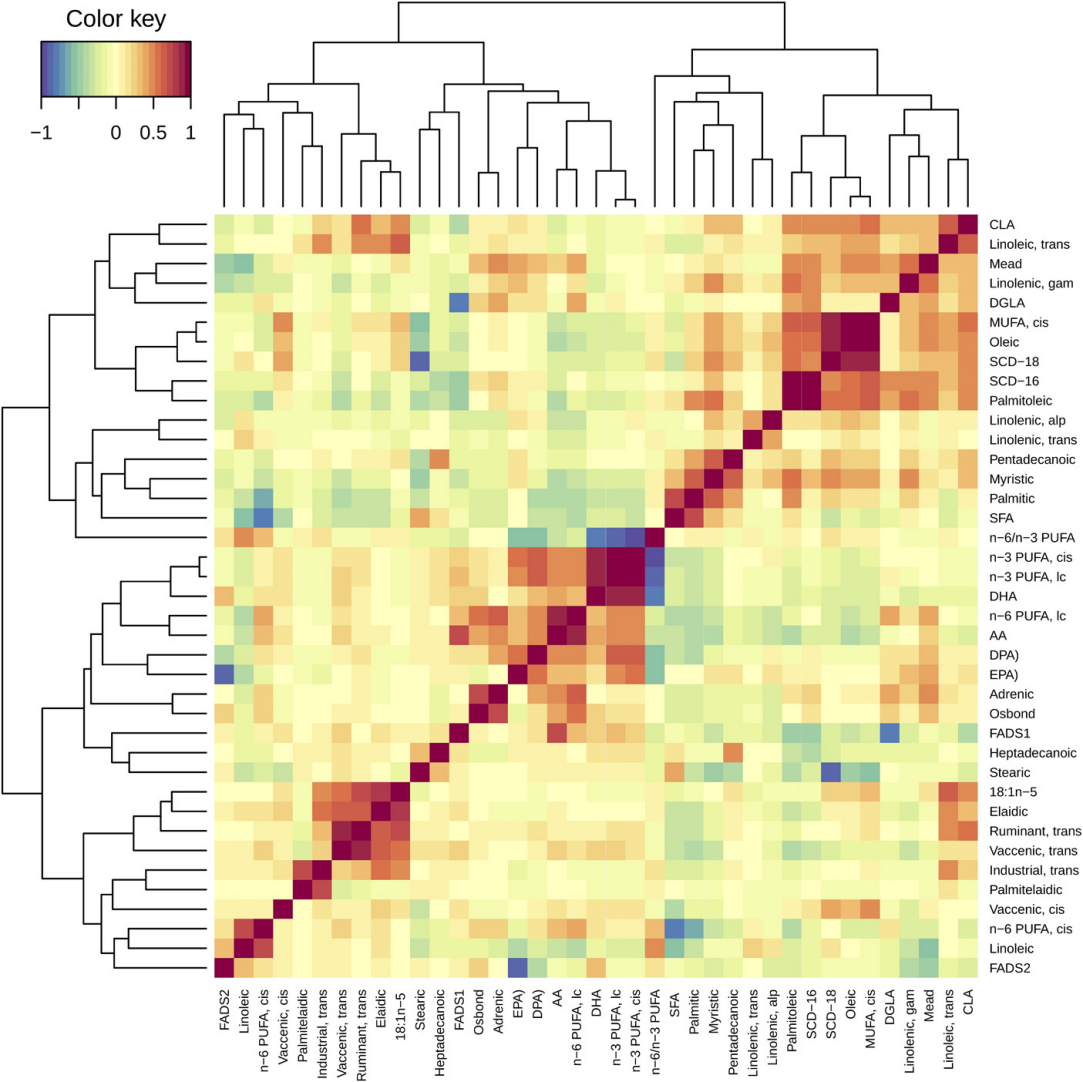
Correlation matrix between the FAs. Correlations between FAs are represented by color squares, bluish for negative correlations, reddish for positive correlations, and yellowish for no or weak correlations. Rows and columns are reordered in clades according to a hierarchical clustering method based on the Euclidean distance. FAs were organized in two major clades, the first including mostly *cis*-n-3 PUFAs, *trans* ruminant and industrial FAs, and desaturation indices, FADS1 and FADS2, while the second clade includes *cis*-MUFAs, SFAs, and desaturation indices, SCD-16 and SCD-18. FAs of the same group are positively correlated with each other, probably due to similar dietary sources. SCD-16 was significantly correlated to most of the FAs associated with BMI, waist circumference, and WHR.

We investigated to determine whether the association between the indicators of obesity and some specific FAs might be influenced by their inter-relationship with other FAs. **Figure 1** shows matrices correlation between the individual FAs, the groupings, and the desaturation indices (color matrix without numeral). SCD-16 showed strong correlation with most of the FAs associated with the obesity indicators, heptadecanoic acid (*r* = −0.361, *P* = 0.000), stearic acid (*r* = −0.280, *P* = 0.000), *cis*-vaccenic acid (*r* = 0.165, *P* = 0.002), γ-linolenic acid (*r* = 0.517, *P* = 0.000), DGLA (*r* = 0.457, *P* = 0.000), EPA (*r* = 0.114, *P* = 0.028), and n-6 PUFAs (*r* = −0.174, *P* = 0.001). We wanted to investigate whether the associations between S-PLFAs and obesity indicators were influenced by the endogenous activity proxied through SCD-16. When further adjusted for SCD-16 (supplementary Table S1), similar associations remained with the few exceptions of total MUFAs, which showed negative association with BMI and waist circumference (both *P* and q-values <0.001), among others.

**Table 4** presents the multiple regressions between desaturation indices and BMI, waist circumference, and WHR. Before correction, all the desaturation indices were significantly associated with BMI and waist circumference, but not with WHR. The associations remained significant and positive for SCD-16 (for BMI, β = 0.034, *P* = 0.001, q = 0.014; and for waist circumference, β = 0.013, *P* = 0.002, q = 0.023) and negative for FADS1 (for BMI, β = −0.036, *P* = 0.001, q = 0.014; and for waist circumference, β = −0.012, *P* = 0.005, q = 0.048) after adjustment for multiple testing.

**TABLE 4.** Multiple regression models for the association between the desaturation indices and obesity indicators in the study population

	BMI				Waist Circumference				WHR			
	β*^a^*	SE	*P*	q	β*^a^*	SE	*P*	q	β*^a^*	SE	*P*	q
Desaturation index 16 (16:1n-7*cis*/16:0)	0.034	0.010	**0.001**	**0.014**	0.013	0.004	**0.002**	**0.023**	1.226	0.791	0.12	0.40
Desaturation index 18 (18:1n-9*cis*/18:0)	−0.024	0.009	**0.009**	0.065	−0.007	0.004	**0.052**	0.22	0.243	0.722	0.74	0.97
FADS1 (20:4n-6/20:3n-6)	−0.036	0.010	**0.001**	**0.014**	−0.012	0.004	**0.005**	**0.048**	−0.804	0.833	0.34	0.71
FADS2 (DHA/EPA)	−0.029	0.010	**0.006**	0.053	−0.009	0.004	**0.032**	0.16	−0.175	0.826	0.83	0.97

a Desaturation index values were log-transformed and Z-standardized for the analysis for better comparison. All the regression models were adjusted for age, alcohol consumption, smoking, energy intake, education, physical activity, menopause, and batch of analysis. *P* or *q* values < 0.05 are shown in boldface type.

## DISCUSSION

This is the first epidemiologic study to report the association between S-PLFA levels and the indicators of obesity in a population of Mexican women. We found that desaturation index, SCD-16, as a marker of endogenous synthesis of monounsaturated palmitoleic acid, was positively associated with BMI and waist circumference. In contrast, SCD-18, FADS1, and FADS2 were negatively associated with BMI and waist circumference. Regarding individual FAs, BMI and waist were negatively associated with heptadecanoic acid and positively associated with EPA and DGLA. WHR was positively associated with the n-6/n-3 PUFA ratio. These findings suggest that specific classes of FAs may be involved in the etiology of obesity and underscore the importance of measuring FA biomarkers for furthering understanding of the role of dietary FAs and metabolism in obesity.

Few epidemiological studies have investigated the relation between individual FAs, desaturation indices, and adiposity. Available epidemiological studies have reported a positive association between palmitoleic acid, DGLA, TFAs and SFAs, SCD16, and indicators of obesity. However, these studies have applied discrepant methodological approaches using, variably, body weight change (17), BMI (21, 29, 30), waist circumference (31), WHR (32), and body fatness (21, 29) as obesity indicators, and as biomarkers of FAs, S-PLFAs (or plasma) (17), whole-blood or erythrocyte FAs (33), and adipose tissue FAs (29), which limit the comparison between studies. In parallel, other studies have assessed dietary intake of FA classes (34), an approach shown to be prone to measurement errors.

In comparison with the European EPIC study for which S-PLFAs are available in a large sample of the population through the same methodology (11), S-PLFA composition in this population of Mexican women was mainly characterized by low levels of n-3 PUFAs and suitably four times higher n-6/n-3 PUFA ratio. This imbalanced n-6/n-3 ratio is in agreement with a study reporting an estimated dietary n-6/n-3 ratio of 16.3 in a nationally representative study in the Mexican population (35), while a ratio of one to four is recommended (36). Second, percentages of ITFAs were more than double in Mexican women compared with EPIC participants (11).

This latter observation is of concern, as industrially produced TFAs from partially hydrogenated vegetable oils induce changes in circulating lipid content, alterations in metabolic and signaling pathways, systemic inflammation, endothelial dysfunction, and insulin resistance and contribute to higher the risk of coronary heart disease and cardiovascular disease (37). Regarding obesity, animal studies reported slight weight gain and abdominal obesity with increasing intake of ITFAs (38, 39), whereas the findings are not supported by epidemiological studies (34). In the present study, we also failed to report a significant positive association between ITFAs and indicators of obesity. The cross-sectional design of our study might explain the lack of an association between biomarkers of TFAs and obesity indicators. Indeed, a prospective evaluation of plasma phospholipid FAs and weight gain in Europe showed a positive association between baseline plasma levels of ITFAs and weight gain in women (17). In addition, the present study population is quite homogenous with respect to BMI and TFA range contents. Additional prospective epidemiological studies are needed to evaluate the relation between individual and total TFAs and obesity in populations with a wider range of BMI and TFA consumption.

Our findings suggest a significant relation between increased endogenous palmitoleic acid synthesis and obesity. While it is not possible in the present cross-sectional design to conclude on any causal relationship between SCD-16 and obesity, numerous epidemiological studies have also previously reported a positive association between obesity and SCD-16 in women, irrespective of their age (21, 30). At a physiological level, the SCD activity is used by the body to convert SFAs to MUFAs to prevent the intracellular accumulation of SFAs susceptible to initiate lipoapoptosis, especially palmitate-induced apoptosis (40). An increased desaturation index measured in plasma phospholipids has been associated with metabolic disorders related to obesity, including insulin resistance, diabetes, cardiovascular diseases, inflammation, metabolic syndrome, and breast cancer (12).

In agreement with previous studies (20), we found dissimilar association with obesity for SCD-16 and SCD-18, which are both expressions of SCD-1 activity. In a cross-sectional study, measurement of SCD-1 activity in liver samples showed that SCD-16 better reflects the expression of hepatic SCD-1 than SCD-18 (41). In fact, in contrast to oleic acid, palmitoleic acid in the diet is low. Therefore, the ratio 18:1n-9/18:0 is susceptible to encompass a large dietary component compared with 16:1n-7/16:0, largely supported by body cell synthesis (42), suggesting that SCD-16 is a more reliable marker of endogenous lipogenesis than SCD-18. Thus, the positive association between SCD-16 and BMI might reflect increased endogenous synthesis of palmitoleic acid, while the inverse association between SCD-18 and BMI might be related to a diet rich in vegetable oils rich in oleic acid.

There is a dearth of evidence on how SCD-16, as a reflection of endogenous synthesis of palmitoleic acid, is affected by diet and hormones. In a controlled cross-over study, a high dietary intake of SFAs has been shown to increase the SCD-16 measured in blood cholesterol esters and phospholipids (42). SCD-1 activity is also activated by high-carbohydrate diets, especially those enriched in rapidly absorbed refined carbohydrates, such as glucose and fructose (43). As a consequence, the high SCD-16 in serum phospholipids that is positively associated with BMI in our population study may be the result of a diet rich in SFAs and carbohydrates, with concomitant increased hepatic desaturation of dietary palmitic to palmitoleic acid. Finally, insulin is an upregulator of SCD-16 activity (44), suggesting that increased SCD-16 associated with increased BMI might reflect an underlying metabolic profile characterized by chronic hyperinsulinemia.

Obesity is characterized by a series of alterations in phospholipid FA content and composition and their endogenous synthesis, resulting in greater susceptibility to inflammation. In obese subjects, serum short-chain MUFAs and SFAs are positively correlated to inflammatory markers and C-reactive protein, while odd-chain and iso-branched chain SFAs showed inverse association (18). Eicosanoids are the products of n-3 and n-6 PUFAs, which include pro-inflammatory eicosanoid mediators from AA, whereas anti-inflammatory eicosanoid products are produced from the n-3 PUFAs (45). Animal studies and in vitro studies indicated that EPA and DHA can hinder the production of an array of inflammatory cytokines, including TNFα, IL-1, IL-6, IL-8, and IL-12 (46). Likewise with inflammation, n-3 PUFAs are, in general, negatively associated with obesity (33). It is therefore intriguing that in our study, we found a positive association between EPA and BMI and waist circumference, despite few studies reporting similar findings (47). However, serum content of EPA and other individual n-3 PUFAs depends on dietary intake, enzyme activity, and the competition in enzymatic conversion between n-6 and n-3. Therefore, the serum n-6/n-3 PUFA ratio, which considers the balance between the two families, might be more informative than individual PUFAs. We found a significant positive correlation between the n-6/n-3 PUFA ratio and WHR in our study. This ratio is exceedingly increased in typical Westernized diets, where it amounts to 20:1 and even more (48), and is positively associated with diverse chronic diseases, including atherosclerosis, obesity, diabetes, and breast cancer (48, 49). Specifically regarding obesity, the mechanism involved in the obesogenicity of an imbalanced n-6/n-3 ratio is not fully elucidated, but Simopoulos (48) suggested multiple pathways involving adipogenesis, lipid homeostasis, appetite regulation, and, most importantly, systemic inflammation. Interestingly, Kaska et al. (50) have previously shown in a population of morbidly obese women that the n-6/n-3 PUFA ratio was positively correlated with C-reactive protein. It can therefore be hypothesized, as our population study is predominantly constituted of obese women, that the positive association found between the n-6/n-3 PUFA ratio and obesity is mediated by inflammatory pathways.

Interestingly, our results showed that odd-chain FAs were negatively associated with obesity. Odd-chain FAs have recently gained interest as potential biomarkers for dairy product intake (51). Pentadecanoic acid and heptadecanoic acid are reported to be negatively associated with several disease outcomes, including diabetes, atherosclerosis, and cardiovascular diseases (52). Our results concur with Kratz, Baars, and Guyenet (53), who reviewed 16 studies and reported that dairy fats might be associated with lower obesity risk, although Mika et al. (54) found no difference in serum levels between individuals with and without obesity. This study suggests that the dietary source of the SFAs might determine their implication in obesity.

The main strength of our study is that it includes data from a country where the incidence of obesity has increased tremendously within a short period of time, escalating from 40% to more than 65% in 40 years (1), suggesting a lifestyle implication in this steady increase. The silhouettes available in our study showed that the majority of the participants were lean during childhood and adolescence and had developed obesity during life course, bringing upfront the implication of the environment and food consumption as promoter of the obesity in Mexico. However, our study is limited by its cross-sectional design. Blood samples and anthropometric measurements were collected together during recruitment, and therefore the association between obesity and FAs found should be interpreted in this specific context. The study was also limited by the lack of information on other biological makers, such as serum triglycerides, C-reactive protein, glucose, insulin, and cholesterol, which could have provided an input to the interpretation of the associations between FAs and obesity. In addition, as mentioned before, our population is homogenous and constituted predominantly of overweight and obese women, and the variability in FA levels in the population is low.

In conclusion, this study showed a positive association between SCD-16 as a proxy for the endogenous production of palmitoleic acid and BMI. In addition, the n-6/n-3 PUFA ratio was positively associated with WHR, suggesting that n-6 PUFAs impact obesity depending on baseline concentration of n-3 PUFAs. Therefore, public health efforts toward adherence to a recommended n-6/n-3 PUFA ratio of one to four might reveal its importance in the fight against obesity. Nevertheless, the causality of the associations found in this study needs to be further investigated. Furthermore, more studies are required to evaluate the relation between obesity and various SFAs with a specific focus on dairy-sourced odd-chain FAs.

## Supplementary Material

Supplemental Data
