# Supplementary material for: Association between serum phospholipid fatty acid levels and adiposity in Mexican women
Source: J Lipid Res. 2017 May 2;58(7):1462–70. doi: 10.1194/jlr.P073643 (PMC5496042; doi:10.1194/jlr.P073643)
Supplement: Supplemental Data [file supp_58_7_1462__index.html]

Association between serum phospholipid fatty acids levels and adiposity in Mexican women — Association between serum phospholipid fatty acid levels and adiposity in Mexican women — Supplemental Data 

# Association between serum phospholipid fatty acid levels and adiposity in Mexican women

## Supplemental Data

- Supplemental Table 1 (.pdf, 195 KB) - Multiple regression models for the association between serum phospholipid fatty acids and obesity indicators additionally adjusted for SCD-16
