# Supplementary material for: Association between serum phospholipid fatty acid levels and adiposity in Mexican women
Source: J Lipid Res. 2017 May 2;58(7):1462–70. doi: 10.1194/jlr.P073643 (PMC5496042; doi:10.1194/jlr.P073643)
Supplement: Supplemental Data [file 10.1194_P073643_jlr.P073643-1.pdf]

Supplementary Table 1: Multiple regression models for the association between serum phospholipid fatty acids and obesity indicators additionally adjusted for SCD-16

	BMI				Waist circumference				WHR			
	**β***	**SE**	**p**	**q**	**β***	**SE**	**p**	**q**	**β***	**SE**	**p**	**q**
**Saturated fatty acids (SFA)**												
14:0 (myristic acid)	-0.004	0.007	0.57	0.87	-0.001	0.003	0.84	0.97	0.801	0.567	0.16	0.47
15:0 (pentadecanoic acid)	-0.002	0.009	0.81	0.97	-0.006	0.004	0.083	0.31	-1.84	0.68	**0.008**	**0.064**
16:0 (palmitic acid)	-0.0003	0.007	0.97	0.98	-0.0005	0.003	0.85	0.97	0.195	0.559	0.73	0.97
17:0 (heptadecanoic acid)	-0.015	0.010	0.12	0.40	-0.013	0.004	**0.001**	**0.014**	-3.36	0.73	**0.000**	**0.0002**
18:0 (stearic acid)	0.033	0.009	**0.000**	**0.0002**	0.008	0.004	**0.025**	0.13	-0.656	0.683	0.34	0.71
**Monounsaturated fatty acids (MUFA)**												
Cis-MUFA												
16:1n-7 (palmitoleic acid)	-0.0001	0.003	0.97	0.98	-0.0002	0.001	0.85	0.97	0.073	0.210	0.73	0.97
18:1n-5	-0.004	0.102	0.71	0.97	-0.001	0.004	0.78	0.97	-0.913	0.793	0.25	0.62
18:1n-7 (cis-vaccenic acid)	-0.031	0.010	**0.002**	**0.023**	-0.012	0.004	**0.005**	**0.048**	-2.010	0.788	**0.011**	0.077
18:1n-9 (oleic acid)	-0.038	0.008	**0.000**	**0.0002**	-0.015	0.003	**0.000**	**0.0002**	-1.135	0.631	0.073	0.28

Trans-MUFA												
16:1n-7/9 (palmitelaidic acid)	0.006	0.008	0.42	0.77	0.001	0.003	0.66	0.95	-0.085	0.610	0.89	0.97
18:1n-9/12 (elaidic acid)	-0.006	0.010	0.53	0.86	-0.0003	0.004	0.94	0.98	-0.167	0.787	0.83	0.97
18:1n-7 (vaccenic acid)	0.001	0.008	0.91	0.98	0.0002	0.003	0.95	0.98	-0.580	0.623	0.35	0.71
**Polyunsaturated fatty acids (PUFA)**												
Cis n-6 PUFA												
18:2n-6 (linoleic acid)	-0.001	0.010	0.93	0.98	0.006	0.004	0.14	0.44	1.790	0.790	0.023	0.13
18:3n-6 (γ-linolenic acid)	0.009	0.009	0.31	0.7	-0.003	0.004	0.39	0.75	-0.650	0.692	0.35	0.71
20:3n-6 (di-homo-γ-linolenic acid, DGLA)	0.028	0.009	**0.002**	**0.023**	0.007	0.004	0.059	0.24	0.069	0.725	0.92	0.98
20:4n-6 (arachidonic acid)	0.002	0.010	0.87	0.97	-0.001	0.004	0.76	0.97	-0.251	0.791	0.75	0.97
22:4n-6 (adrenic acid)	-0.016	0.010	0.11	0.39	-0.006	0.004	0.13	0.42	0.640	0.779	0.41	0.76
22:5n-6 (osbond acid)	-0.035	0.010	**0.001**	**0.014**	-0.012	0.004	**0.004**	**0.042**	0.628	0.820	0.44	0.79
Trans-n-6 PUFA												
Conjugated linoleic acid (CLA)	-0.002	0.009	0.81	0.97	-0.0004	0.004	0.99	0.99	-0.264	0.696	0.70	0.97

18:2ct, 18:2tc, 18:2tt (trans linoleic acid)	-0.009	0.008	0.26	0.64	-0.002	0.003	0.56	0.87	-0.169	0.648	0.79	0.97
Cis-n-9 PUFA												
20:3n-9 (mead acid)	-0.004	0.009	0.63	0.91	-0.005	0.004	0.20	0.54	-0.626	0.714	0.38	0.74
Cis-n-3 PUFA												
18:3n-3ccc (α-linolenic acid)	-0.013	0.011	0.22	0.56	-0.004	0.004	0.32	0.7	-0.552	0.834	0.51	0.86
20:5n-3 (eicosapentaenoic acid, EPA)	0.023	0.010	**0.029**	0.15	0.006	0.004	0.18	0.51	-0.567	0.823	0.49	0.85
22:5n-3 (docosapentaenoic acid, DPA)	-0.007	0.010	0.51	0.86	-0.001	0.004	0.80	0.97	-0.031	0.784	0.97	0.98
22:6n-3 (docosahexaenoic acid, DHA)	-0.003	0.010	0.75	0.97	-0.003	0.004	0.41	0.76	-1.096	0.784	0.16	0.47
Trans-n-3 PUFA												
18:3n-3cct, ctt, ttt (trans α-linolenic acid)	0.0006	0.011	0.95	0.98	0.003	0.004	0.54	0.86	0.794	0.833	0.34	0.71
**Groupings**												
Total SFA	0.020	0.009	0.032	0.16	0.004	0.004	0.31	0.7	-0.388	0.711	0.59	0.89
Total cis-MUFA	-0.038	0.007	**0.000**	**0.0002**	-0.016	0.003	**0.000**	**0.0002**	-1.359	0.598	**0.024**	0.13

Total trans ruminant fatty acids	-0.003	0.009	0.71	0.97	-0.002	0.004	0.62	0.9	-0.786	0.713	0.27	0.66
Total trans industrial fatty acids	-0.0007	0.009	0.94	0.98	0.0005	0.004	0.89	0.97	-0.112	0.729	0.88	0.97
Total cis n-6 PUFA	0.005	0.009	0.57	0.87	0.007	0.004	0.076	0.29	1.765	0.736	**0.017**	0.11
Total long-chain n-6 PUFA	0.009	0.010	0.39	0.75	0.0006	0.004	0.88	0.97	-0.109	0.785	0.89	0.97
Total cis n-3 PUFA	0.0005	0.010	0.96	0.98	-0.002	0.004	0.68	0.95	-1.029	0.786	0.19	0.52
Total long-chain n-3 PUFA	0.002	0.010	0.84	0.97	-0.001	0.004	0.77	0.97	-0.955	0.783	0.22	0.56
Ratio PUFA n-6/n-3	0.002	0.010	0.88	0.97	0.004	0.004	0.32	0.70	1.66	0.81	**0.041**	0.19

SE, standard error

** Fatty acids values were log-transformed and Z-standardized for the analysis for better comparison. All the regression models were adjusted for age, alcohol consumption, smoking, energy intake, education, physical activity, menopause, batch of analysis and SCD-16*
